# Supplementary material for: The Protocol for: Do peers enhance behavior change in group motivational interviewing? A translational clinical trial investigating brain synchrony in underage emerging adults during an fMRI hyperscanning task and association with alcohol use reductions
Source: PLoS One. 2026 Jun 11;21(6):e0349575. doi: 10.1371/journal.pone.0349575 (PMC13257965; doi:10.1371/journal.pone.0349575)
Supplement: S2 Table — (DOCX) [file pone.0349575.s002.docx]

# Minimal Risk Protocol Template (IRB Approved Protocol)

1. Protocol Title

Partnering to Enhance Emerging adults' Response to programs (PEER)

Grant title (not participant-facing): Do peers enhance or detract progress in group MI? A look into emerging adult brain and behavior

1. Objectives

This is a within-subjects design measuring: (1) youth brain response to their own change language during group motivational interviewing (MI), (2) youth brain response to peer-peer dyadic exchanges during group MI (specifically, to peer language), and (3) how brain response is associated with youth behavior change at 3-, 6-, and 12-months post-treatment.

1. Background

Emerging adulthood is a unique developmental period [69-71]. In the US, emerging adults under the legal drinking age (underage emerging adults; U-EA; ages 18-19) are expected to separate and individuate, and in doing so, transition to more “adult” roles (e.g., going to college/obtaining more advanced professional training; shifting into independent living situations; routinely attending social events without parental supervision) [72] that increasingly include alcohol [1, 71, 73]. Yet, among U-EAs, the brain regions responsible for weighing consequences, judging costs and benefits, and self-regulating, particularly in the context of decision-making around drinking, are still very much in development [70, 74, 75]. This is important because recent data indicate that alcohol use during this final stretch of neurodevelopment (up until age 25) may be neurotoxic [8, 9]. Meaning, that drinking in hazardous ways, such as heavy episodic use (>4/3 standard drinks for males/ females) [76-78], may disrupt U-EAs’ neuro-developmental trajectory by elevating risk for sustained patterns of alcohol use and hazardous outcomes as they transition into adulthood [79] [8, 9].

**Hazardous or at-risk alcohol use during adolescence**. Addiction terminology continues to be optimized to discuss risky alcohol use in non-stigmatizing ways [80-82]. The PI’s recent work [83] along with the late Dr. Saitz, recommended “hazardous or at-risk use” to represent alcohol use that increases the risk for health consequences [80]. PI Feldstein Ewing and her team have been advocating for terms and frameworks that more accurately capture the differential nature of alcohol use in youth [2, 48]. To this end, clinical categorizations of alcohol use disorder (AUD) as defined through standard mental health diagnostic criteria [e.g., DSM; 84] tend to be a poor fit with patterns of drinking observed in youth [85]. Our team has thus focused examinations of harmful alcohol use (and its resolution) in this age group around metrics of interference in functioning (defined and measured as “hazardous drinking”), to represent the manifestation of the adverse impact that drinking can have on social/peer, school/academic, work, and family spheres among U-EA [2, 86].

**What makes the problem especially challenging**. Not only do U-EAs engage in heightened hazardous drinking [10], but U-EAs engaged in hazardous drinking are unlikely to seek, receive, or complete indicated alcohol intervention [11]. In turn, improving the effectiveness, particularly of brief interventions, is requisite to intercepting this trajectory of alcohol use and related risk before U-EAs reach adulthood [12, 13]. Paralleling our own findings with U-EAs [2, 3, 48, 59, 87-92], recent reviews indicate that among the youth alcohol interventions with the strongest empirical support [e.g., motivational interviewing [MI; 44], cognitive behavioral therapy (CBT), and family based interventions (FB)], MI consistently rises to the top in terms of brief interventions, but findings still leave substantial room for improvement [38, 93], with effect sizes for youth trailing behind those observed among adults [47] [48] [38, 49]. Further, with notable exceptions [e.g., 57, 58], much of the MI mechanistic research has primarily been examined within individualized contexts [50-56]; work examining mechanisms of group-based MI particularly with U-EA has only just begun [57, 58].

**Relevant factors**. Our team proposes that three factors may be key to improving MI outcomes with U-EA:

(1) **The nature of the developing U-EA brain**. The developing U-EA brain is now recognized as having unique function, structure, and connectivity [70, 71, 94]. Our own data support this (Figure 1), wherein we have found a distinct pattern of neural responses, which we believe subserve a developmentally-specific pattern of neurocognitive response to MI interventions among youth, largely localized to default mode network (DMN) regions [(precuneus; posterior cingulate cortex (PCC)] [95] [2, 3, 59-61]. Across our independent youth translational (brain:behavioral) studies (total estimated N=1136), our team has found that youth who receive our brief (1-2 session) MI interventions significantly decrease their alcohol use and other health risk behavior across follow-ups (1 month through 12 months) [2, 3, 59-61] [87, 90, 92, 96-101]. Our studies also indicate that during our functional magnetic resonance imaging (fMRI) task designed to examine within-session active ingredients (client language; therapist language), youth display a distinct neurodevelopmental pattern, with greater blood oxygenated level dependent (BOLD) response in regions central to self-reflection/contemplation and self-awareness (DMN; e.g., precuneus, PCC) [2, 3, 59-61]. This response is distinct from the pattern of mesocorticolimbic reward response observed among adults with parallel fMRI tasks [60], and the nature of adult neural patterns of AUD more broadly [102-105]. Together, these findings support a relationship for neural response to within-session active ingredients in DMN that are significantly associated with post-intervention behavior change among youth [2, 3, 59-61]. DMN is relevant, as the literature highlights that it is central to mentalizing, past/future thinking, emotional, and self-referential processing [106-108]. These aspects of neurocognition are already elevated in the U-EA developmental period due to the natural escalation of self-examination that occurs in this window [109]. As DMN has an integral role in mentalizing and self-reflection, it also makes sense that DMN is a natural locus for neural response and successful behavior change during MI as well [110] [111] [2]. In addition, these data underscore the need to examine other widely-used MI-based interventions, to determine to what degree other modalities, such as group MI, may also enhance DMN response among this high-need population.

(2) **The role of peers in U-EA alcohol use**. One steadfast factor through decades of youth alcohol-related risk research is the role of peers [13-17]. During this stretch, U-EAs naturally spend less time with family, and more time with peers. This dynamic often facilitates a rise in U-EAs’ opportunity to experiment with more frequent (and heavier) drinking episodes [1, 48, 112-114]. One of the more concerning aspects of this ascent in U-EA hazardous drinking is that most U-EA do not perceive their alcohol use as hazardous [71]. Instead, many U-EA predominantly report positive social experiences related to their drinking (e.g., feeling more accepted at parties; having more fun in social gatherings; being less socially anxious) [1, 114]. As presented in our recent Lancet: Child & Adolescent review [1], the U-EA developmental period is demarcated by “social attunement,” manifesting via a surge in the need to harmonize with U-EAs’ peers and their broader social environment. Empirically, neurodevelopmental data support that the U-EA brain shows enhanced capacities for peer-related social monitoring and detection [18-22], differentially activating in the context of peers [18-22], primarily within a network we call the “social cognition network,” [1] which includes medial prefrontal cortex (mPFC) [62-65], superior temporal sulcus (STS) [62, 63, 65, 66], and temporal parietal junction (TPJ) [29, 62, 63, 67, 68] (Figure 2). This neural activation is followed by U-EAs capacity to quickly modify their behavior in order to be in sync with peers [1]. Important in this equation is U-EAs’ perception of peer substance use, which is associated with both current U-EA use and their future use progressions [1].

(3) **Peer behavior in the context of group-based interventions**. The role of peer behavior is salient, as one of most widely used platforms for U-EA intervention service delivery is group-based formats [35-39]. Despite the clinical ubiquity of group-based interventions for U-EA, research on mechanisms and outcomes of group interventions with youth has been limited [40]. There are notable advantages to the group format (e.g., efficiency, cost-effectiveness, youth comfort with peer groups) [100]. Yet, there are also reasons for concern, as early seminal work found “iatrogenic outcomes,” wherein when youth were grouped together, youth who displayed greater positive reactions to peer-to-peer rule-breaking conversations showed significantly higher alcohol, cannabis, and tobacco use at follow-up, even when those youth had minimal use at study entry [41-43]. Equally important, these studies also indicate that the powerful influence of peers may hold promise for being harnessed in positive directions, with the potential to reduce youth health risk and/or promote prosocial behavior [41-43]. In turn, the degree to which having young peers together, along with the nature (and mechanisms) of those peer-to-peer exchanges within group-based interventions, remains under-examined.

**How this approach expands the standard paradigm**. Via PAR-21-280 “Dyadic Interpersonal Process and Biopsychosocial Outcomes” and grounded in the NIH-funded youth translational approach that our team has used for 15 years, we plan to utilize functional magnetic resonance imaging (fMRI) to disaggregate the interaction between U-EA brain response among previously unfamiliar group MI dyadic peers and subsequent intervention outcomes (alcohol use days; hazardous alcohol use), in the underexamined, but widely utilized group therapy context. We believe that the key to developing more successful interventions for youth engaged in hazardous drinking is through applying a translational perspective to understand basic brain factors that influence U-EA intervention response in group MI contexts.

**Why MI?** Originally developed and validated as an individual-level intervention [44, 45, 115], MI has been widely deployed in direct practice settings as a group-based intervention for over two decades. Yet, the empirical research on intervention outcomes with group MI, particularly among youth recipients, has lagged behind individual-level evaluations [89]. Importantly, a handful of teams, including our own, have evaluated group MI across a wide range of health behaviors (including, but not limited to: alcohol and other substance use; STI/HIV risk behavior). Overall, findings largely support group MI as a modality that effectively generates behavior change in the targeted behavior [57, 58, 89, 90, 96, 98, 99, 116-136]. An even smaller handful of investigators have taken this work a step further by evaluating mechanisms of group MI, including within-session factors that impact group MI outcomes with youth. Collectively, these findings suggest that among young participants, positive statements in favor of change (change talk) during group MI have been associated with significant improvements in post-treatment alcohol-related behavior [57, 58, 137], in contrast with negative statements in favor of staying the same (sustain talk), which have been associated with poorer post-treatment outcomes. We could only find one peer-reviewed published study looking at group MI delivered in a peer-dyad format; in the one we found, effect sizes for alcohol use reductions were 3 times larger in the peer-dyad group MI than for the individually-delivered format [138]. Yet, we found no studies looking at the impact of the peer-peer exchange on brain response. In other words, group MI is widely used, but we know much less about how (and why) it works, and how (and when) it does and does not facilitate youth behavior change. Most importantly, we know even less about how interactions with same-age peers may influence both brain and behavioral response.

**The merit of examining reciprocal peer-peer dyadic interactions in group MI with young people**. Not only does the peer-dyad format appear to be a particularly robust way to deliver group MI in this age group [138], but there is an emerging literature on the impact of positive peer feedback in the context of group therapy more broadly [139, 140]. Yet, we could find no peer-reviewed studies evaluating the nature of peer-to-peer exchange in group MI and how it might impact intervention outcomes for U-EAs. The relevance of this question is that the developmental neuroscience literature robustly reflects that peers hold higher neural salience during this developmental window, as evidenced by youths’ differential neural response to conditions with real and/or simulated peers [18-22], even when those peers were not friends [23], and particularly in the context of alcohol [24-26], with neural activation largely found in the social cognition network [1]. While much of the literature has focused on the potential negative impact of peer feedback on youth neural activation and related behavior, studies are now recognizing that peers concomitantly activate positive (prosocial) neural and behavioral responses in this age group [27-33]. Equally important, recent studies have highlighted the myriad adaptive aspects of this enhanced period of neurosocial responsiveness; here, the nature of the developing brain can be viewed as having unique cognitive assets, including heightened social awareness, and enhanced facility to subsequently modify behavior [1]. In sum, not only could we find limited studies on the neural and behavioral response of U-EA in the potentially highly impactful group MI peer-dyad format, we could also find no published literature examining how being in a group context with other peers may enhance youth brain response in group interventions, or how those neural peer feedback experiences may impact behavior change post-group MI [141].

**Significance**

**Why testing this hypothesis or solving this problem is important**. (1) Public health significance. Alcohol continues to be the top substance used by U-EA [4, 5] and 2021 data reflect that youth drinking is on the rise in the US [5]. In addition to alcohol-related negative health, safety, and neurodevelopment sequalae [8, 9] [12, 13], only a fraction (<6%) of U-EA engaged in hazardous drinking receive intervention [142]. Among those who do, behavior change in this age group still remains modest [52, 143]. As one example, meta-analyses examining MI have shown that effect sizes for MI treatment outcomes are less robust for youth (d = 0.17) [144] [56] as compared with adults (d = 0.77) [45]. In turn, although the use of MI is widespread, we are still at the precipice of understanding the role of within-session mechanisms, particularly for U-EAs, for the group MI modality [145]. (2) Closing the gap between science and practice in U-EA group interventions. Given their brevity, low cost, and ease of dissemination [143], the group modality is already widely used to deliver alcohol intervention with U-EAs. Even with the research around how potential peer-peer iatrogenic outcomes during group interventions may occur with young people [41-43], we have a limited understanding of how peers, and specifically peer-peer interactions may *enhance* intervention outcomes within the group MI platform [141]. This is timely, as there has been a surge of publications within the developmental neuroscience literature around the salience and importance of peers in terms of neural response, behavioral risk, and prosocial choices [1]. In sum, we could find no peer reviewed literature examining the degree to which peer-peer dyadic exchanges within the context of real-world live intervention receipt may impact both brain and behavioral response for U-EA. This study will fill that gap by examining the nature of peer-peer dyadic exchanges of previously unfamiliar dyads within group MI. Here, we will carefully evaluate how positive peer language (peer-generated change talk) impacts neural response, and how neural response may predict group MI outcomes. This is critical to address the gap around how and why group MI may (or may not) work in this age group, and will generate crucial next step data to meaningfully move the needle on enhancing alcohol-related interventions and related outcomes in this high-need age group.

**Why fMRI? The added benefit of fMRI to guide and improve treatments for U-EAs**. As with others [88, 146], our ultimate goal in this work is to use a much more precise instrument (here, fMRI) to enable a highly sensitive and specific view into the nature of U-EA brain response during real-world behavioral interventions. Because their brains are still very much in development, as with our other translational (brain:behavioral) studies, this approach illuminates how we can more optimally target and improve clinical approaches for this age group. For example, in prior studies, when compared with adults, our team has learned that youth brains respond in very different regions and at inverse time points (e.g., DMN vs. mesocorticolimbic regions; during change talk as compared with sustain talk) [48, 60, 61]. These critical translational data support that behavioral alcohol interventions that feature and/or focus on the context of reward (e.g., contingency management), may be less impactful with younger age groups, who have less brain response in reward areas. In this proposal, in the novel context of group MI, we will evaluate areas of U-EA neural response; what we find will alert us to which regions require more attention during articulated interventions to maximize U-EA response during group-based behavioral interventions. For example, if we observe neural response in the TPJ during positive peer statements (peer-generated change talk), then we will undertake modifications to our group-based clinical approaches. Concretely, that might take the form of developing new clinical avenues to have U-EAs engage in heightened amounts and/or various types of positive/prosocial peer-peer exchanges during the group MI, and/or other neurocognitive processes subserved by the TPJ [63, 147]. In addition, in our other studies, we have observed minimal youth neural response when therapists utilized confrontational language during individual-level behavioral interventions; further, neural response during confrontational language was not associated with youth behavior change [2]. From these data, we have been sharing with pediatric practitioners and clinicians the importance of not using confrontational language with young people, even when operating in very time-limited contexts [2, 88, 148]. Data from this study is essential to allowing us to help reduce drinking in a high-need and underserved community of young people.

1. Study Design

This is a within-subjects design measuring: (1) youth brain response to their own change language during group MI, (2) youth brain response to peer-peer dyadic exchanges during group MI (specifically, to peer language), and (3) how brain response is associated with youth behavior change at 3-, 6-, and 12-months post-treatment. Within this study, two U-EAs engaged in hazardous drinking will be scheduled to attend a “Participation Day,” comprised of a baseline behavioral assessment (which they will complete alone), a single 1-hour session of virtual group MI (completed together), and to ensure maximum salience of the peer dyadic experience, the fMRI scanning protocol on the afternoon of the same day (completed simultaneously). To determine how these factors relate to alcohol-related behavior change, youth will complete behavioral follow-ups at 3-, 6-, and 12-months post-intervention.

1. Study Population
2. Number of Subjects

N = 248 youth.

1. Inclusion and Exclusion Criteria

Inclusion criteria

For inclusion, youth must be: (1) 18-19 years of age; (2) agree to be contacted for the 3, 6, and 12 month follow ups; (3) provide fully informed consent.

Exclusion criteria

With respect to the fMRI component, we have attempted to maintain tight control over factors that may increase error variability and that maximize protection for our participants. Thus, our exclusion criteria are: (1) left-handed; (2) evidence of brain injury/illness and/or neurological, neurodevelopmental disorder including psychosis and related medications (e.g., antipsychotics; neuroleptics); (3) loss of consciousness ≥ 2 minutes; (4) other fMRI contraindications (e.g., unremovable metal on/in body, pregnant)

1. Vulnerable Populations

This study will not enroll any vulnerable populations.

1. Setting

We will employ community and campus recruitment methods across the greater Dallas, Texas metro area to recruit a sample with sufficient hazardous drinking to assess mechanisms underlying behavior change within this group MI intervention. In-person study procedures (Participation Day) will take place at the University of Texas Dallas (UTD) in private, dedicated rooms in the Center for BrainHealth and adjacent spaces assigned to the UTD team for this purpose. Follow ups (at 3-, 6-, and 12-months) will be conducted virtually using a secure online data collection system (REDCap), and will include a brief online or telephone interview with UTD project staff.

1. Recruitment Methods

We will recruit trial participants via general community outlets (e.g., fliers) posted in the Dallas

metro area with high U-EA traffic (e.g., coffee shops), through social media recruitment

approaches that have been effective for our team with this age group (e.g., Facebook;

Instagram; Snapchat), and through UTD campus recruitment (e.g., classroom sign-ups). This approach follows our >15 years of NIH-funded prevention intervention work with youth. Consistent with our prior underage emerging adult (U-EA) substance use studies [2, 3, 59, 153], U-EA interested in learning more about participating will call in to our program line to learn more about the opportunity to participate (or receive a phone call or text, if they put their name on a sign-up sheet or scanned a QR code). Trained study staff will inform U-EA that PIs Feldstein Ewing and Filbey are developing programs to encourage healthy behavior for individuals their age. U-EA will be informed that eligible youth will be invited to complete a number of study activities, including brain imaging (fMRI), questionnaires, and participation in a health program session with another individual their age. U-EA will be told that participation involves discussing sensitive topics such as alcohol and other substance use (which are illegal for individuals <21), and related health and health risk behaviors (e.g., sexual activity). Study staff will be explicit that participation is voluntary and U-EA can opt out at any time without repercussion. Consistent with our prior NIH and IRB-approved protocols, interested U-EA will be screened for eligibility by our study staff prior to completing consent materials. Eligible U-EA will be invited to complete consent forms via REDCap, which we have found increases accessibility in the consent process. Specific eligibility criteria will not be shared with participants to further protect participant privacy.

Participant compensation

Monetary Compensation

| Baseline  Participation Day  (In-Person) | 3-Month  (Electronic) | 6-Month  (Electronic) | 12-Month  (Electronic) | Total |
| --- | --- | --- | --- | --- |
| $100.00 +  $20 travel | $30 | $35 | $40 | $205.00 +  $20 travel |

SONA Compensation Schedule

| Baseline  Participation Day  (In-Person) | 3-Month  (Electronic) | 6-Month  (Electronic) | 12-Month  (Electronic) | Total |
| --- | --- | --- | --- | --- |
| 5 SONA Credits +  $20 travel | $30 | $35 | $40 | 5 SONA Credits +  $105.00 +  $20 travel |

Participants will receive compensation in the form of GalaxyPay, a pre-paid card similar to a VISA debit card. Consistent with our prior NIH-funded work with youth, youth will receive $100 for completing the Participation Day (in lieu of monetary compensation for the participation day activities, students may opt to receive 5 SONA credits to fulfill certain class requirements), plus an additional $20 to help facilitate travel to the participation site. Youth will receive $30 for the 3 month follow-up, $35 for the 6 month follow-up, and $40 for the 12 month follow-up. In sum, youth can receive a possible total of $205 for participating in all study time points, plus the additional $20 in transportation offset for the in-person visit. If participation is terminated by the participant or the research team before completion of the study, the participant will be compensated at a rate of $20 (or 1 SONA credit during participation day) per hour, not to exceed the total amount listed above for the applicable time point.

1. Consent Process

Prior to beginning any study procedures, trained study staff will inform U-EA via phone that PIs Feldstein Ewing and Filbey are developing programs to encourage healthy behavior for individuals their age. U-EA will be informed that eligible youth will be invited to complete a number of study activities, including brain imaging (fMRI), questionnaires, and participation in a health program with another individual their age. U-EA will be told that participation in this project involves discussing sensitive topics such as alcohol and other substance use (which are illegal for individuals <21), and related health and health risk behaviors. Study staff will be explicit that participation is voluntary and U-EA can opt out at any time without repercussion. Consistent with our prior NIH and IRB-approved protocols, interested U-EA will be screened for eligibility by our UTD study staff prior to completing consent materials. The staff member will be explicit that participation is completely voluntary, meaning that participants can decide to opt out of the research protocol at any time they wish without repercussion, and that participants can always skip questions that they do not feel comfortable answering. Specific eligibility criteria will not be shared with participants to further protect participant privacy.

Study team members will check in with participants prior to beginning the baseline procedures to ensure that participants understand the study details and their rights as participants. Research staff will reiterate the voluntary nature of all aspects of the study, and participants’ right to refuse to answer any questions with which they are uncomfortable and/or to withdraw from the study at any time without any type of recrimination.

Eligible U-EA will be invited to complete consent forms on paper during their participation day, which we have found increases accessibility in the consent process. This system allows the participant to review the entire consent form, discuss the study and have questions answered with a UTD study team member, allows UTD study team member to ensure participants understand the consent form, and complete the form if they consent to participate.

1. Procedures

**Participation Day Procedures (#1-3 below)**. Our UTD team will keep a rolling list of eligible participants. After two same-sex U-EA have been deemed eligible and consented, our team will contact youth to ensure that there is no change in their eligibility (if more than 30 days have passed since screening) and they will then be scheduled in dyads for “Participation Day” activities, which include the baseline assessment (which they will complete alone), the group MI (completed together virtually with a URI study counselor), and the scan (completed simultaneously). We will ensure that U-EA are able to participate on the same day, including completing the baseline assessments at the same time, the group MI together, and then the scan simultaneously on the same afternoon. All intervention groups will be stratified by biological sex (e.g., male, female). This is commensurate with our approach with other group MI protocols with this age group [97-99]. To improve accessibility, all Participation Day components, including the assessment and the group MI, will be delivered in private, dedicated rooms in the Center for BrainHealth and adjacent spaces assigned to our UTD team for this purpose.

- - 1. Biohazards and TimeLine Follow Back.

Primary Measures. In line with our prior studies [2, 3, 59-61], at the start of the Participation Day, youth will be breathalyzed using a BACtrack S80 Professional Breathalyzer to ensure BAC=0. Consistent with the PI’s prior translational work [2, 3, 59-61], all participants will be required to abstain from alcohol and other substances for 24 hours prior to the scan. Upon arrival to the Center for BrainHealth, youth will be screened to assure the absence of fMRI contraindications (no metal in body; not pregnant as verified by urine testing for all female participants; breath alcohol = 0). Only approved youth will be scanned. Our primary outcomes include two variables: alcohol use days and hazardous drinking. We will utilize the TimeLine Follow-Back [TLFB; 172], a calendar-based measure which provides quantity and frequency of past 30 day substance use (including alcohol use days), and has been validated on internet-based platforms [173]. This measure will also provide rates of cannabis and other substance use (e.g., e-cigarette/vaping; prescription opioid use). The second primary outcome of hazardous drinking will be will be assessed following the virtual group MI session via Rutgers Alcohol Problems Index [RAPI; 174].

- - 1. Virtual Group MI Session. In line with our previous group MI approaches to reduce alcohol and related health risk behavior with this age group, all participants will receive 1 60-minute group MI session conducted virtually by a graduate Clinical Psychology PhD student or postdoctoral researcher affiliated with PI Feldstein Ewing’s ANCHoR team and will proceed according to our established group MI manual for U-EA alcohol use [89]. This group MI aims to introduce a conversation about alcohol use and the personally experienced consequences of hazardous drinking [2, 87, 90, 92, 96-100]. Group MI interventionists will conduct the MI in an MI-consistent manner, meaning that they will be open, strength-based, affirming, non-judgmental, and empathic, with a goal of reducing resistance and highlighting ambivalence around hazardous drinking to foster and support U-EAs’ intrinsic motivation for behavior change.

Following our empirically-supported approach for MI targeting alcohol use with non-treatment-seeking youth, the group MI will start with an open-ended exploration of dyad’s hazardous drinking behavior via eliciting the dyad’s stories about their alcohol use. Following this open-ended exploration, group MI interventionists will guide the youth through a values clarification task [186]. The remainder of the group MI will focus on providing age-matched norms from this geographic region (Texas), and an averaged overview of personalized feedback regarding the dyad’s alcohol use (as derived from their baseline assessment data). Following this, the group MI interventionist will actively and collaboratively engage the dyad in the development of discrepancy (exploring how their drinking fits with their immediate and long-term goals), the identification of high-risk situations (e.g., sports events; going to parties; hanging out with certain friends who tend to drink heavily) and triggers for drinking (e.g., feelings of sadness; anxiety; celebration), and an exploration of strategies to manage those risks and triggers to help the dyad reduce their hazardous drinking in those contexts. The ultimate goal of the group MI session is to engage the dyad in a thoughtful conversation about their hazardous drinking and the implications that their hazardous drinking may have on their lives, with an eye to bolstering and supporting the dyad’s inherent drive for behavior change. The group MI interventionist will close with a summary of the session and a broad and non-judgmental query of how the dyad would like their alcohol use to be in the near future (e.g., “Tell me how you would like your drinking to look when our team talks with you in 3 months.”)

Assurance of Intervention Fidelity. To ensure intervention integrity and fidelity, all group MI sessions will follow our group MI manual [89]. This group MI was developed based on the PI’s clinical work with youth engaged in alcohol and other substance use and continues to be updated in line with recent publications in the MI field [57, 58, 89, 90, 96, 98, 99, 116-136]. The PI is a recognized expert in training and conducting MI and group MI with youth. In line with her previous NIH-funded studies, she will train each group MI interventionist via the following 5 steps. Group MI interventionists will: (1) receive didactic training on the theories behind group MI, (2) be required to read the group MI study manual, (3) pass a knowledge test to evaluate their grasp of the concepts within and behind group MI (e.g., with a pass rate of at least 80% on the fidelity checklist and 100% adherence to essential therapeutic elements), (4) watch the PI conduct a pilot group MI and discuss the details of the session, and (5) conduct two pilot group MIs, which will be observed by the PI who will assess the group MI interventionist’s proficiency in the manualized group MI intervention. All sessions will be audio-recorded for the purposes of systematic supervision, allowing PI Feldstein Ewing to ensure fidelity and prevent therapist drift. If a group MI interventionist is found to have lower levels of intervention integrity or significant drift, the PI will provide the group MI interventionist with feedback and training until they are back on track.

- - 1. Baseline Behavioral Assessment. Our team has moved to REDCap for our current NIH-funded studies [2, 169], which has gained support as a user-friendly, reliable, and secure way to obtain survey data, including health risk behavior with this age group [170, 171]. Each member of the U-EA dyad will complete behavioral assessments using REDCap via lab computers in separate rooms after their participation in the virtual group MI. All questionnaires will be self-reported from the participant. All measures are taken by the participant with no assistance from study personnel.

Primary Measures. Our Demographic questionnaire collects age, self-identified gender, race/ethnicity, education, income, parent education, family income, and related information. The RAPI will be during this baseline behavioral assessment to collect hazardous drinking patterns. In terms of relevant youth and peer factors that may be salient to intervention outcomes, U-EAs will complete the UCLA Lonelinesss Scale, the personal responsibility subscale of the Psychosocial Maturity Inventory [176], the Perceived Discrimination Scale, the Pittsburgh Sleep Quality Index, and the Prosocial Subscale from within the Strengths and Difficulties Questionnaire (SDQ) [178] [179, 180]. Additional health risk behavior will be evaluated with the Sexual Behavior Questionnaire. The SBQ will be a requirement due to the co-occurrent behavior that could stem from alcohol use. We will evaluate alcohol use disorder symptoms via the DSM-5 alcohol use disorder criteria [84], and assess cannabis use impairment and symptomatology via the Cannabis Problems Questionnaire and the DSM-5 cannabis use disorder criteria. Immediately post-scan, we will query youth’s level of agreement with the presented statement [182] presented in the fMRI (NILE) paradigm via likert scale.

- - 1. Neuroimaging Session. We will conduct the fMRI session in the afternoon of the “Participation Day” to maximize salience of the peer interaction from the group MI. Between the virtual group MI and scan session, in line with our previous youth translational approaches [2, 3, 59-61], the PI’s team will capture audio recordings of salient peer change talk from the peer health session to integrate into the dyads’ individualized fMRI task (see Figure 6 and our team’s coding experience for details). Scanning will take place at the Center for BrainHealth.

The MRI simulator is available to help participants become acclimated to the scanning environment prior to MRI, if desired. Once a participant has entered the bore of the actual MRI, there will be 2 5-minute runs of resting state with 10 second break in between runs (in line with the current ABCD Study protocol [187]), lastly they will have a high-resolution anatomical scan followed by the fMRI task (Figure 6). Back-projected stimuli will be presented on a visual display at the back of the MRI bore and viewed with a mirror mounted on the head coil. Participants will wear MRI-compatible electrostatic ear bud headphones to hear all auditory stimuli. Foam padding will be used to constrain head motion. Data will be acquired on a Siemens 3 Tesla Prisma scanner (Siemens Medical Solutions, Erlangen, Germany) with a Siemens 64-channel head coil. A high-resolution, T1-weighted anatomical scan will be collected for alignment and normalization of functional images (176 slices 1mm isotropic, matrix = 256x256, TR/TE/TI = 2500/2.88/1060 ms, flip angle = 8°, pixel bandwidth = 240Hz) for each participant. The whole-brain fMRI task scan will be acquired using a T2*-weighted echoplaner (EPI) sequence (2.4mm isotropic matrix = 90x90, TR/TE=800/30 ms, flip angle = 52°, field of view = 216 mm, slices = 60) such that the middle volume will be aligned with the subject’s inter-commissural line (AC-PC). A pair of reversed phase encoded field maps will be acquired to enable distortion correction. Parameters will optimize BOLD quality while maximizing brain coverage.


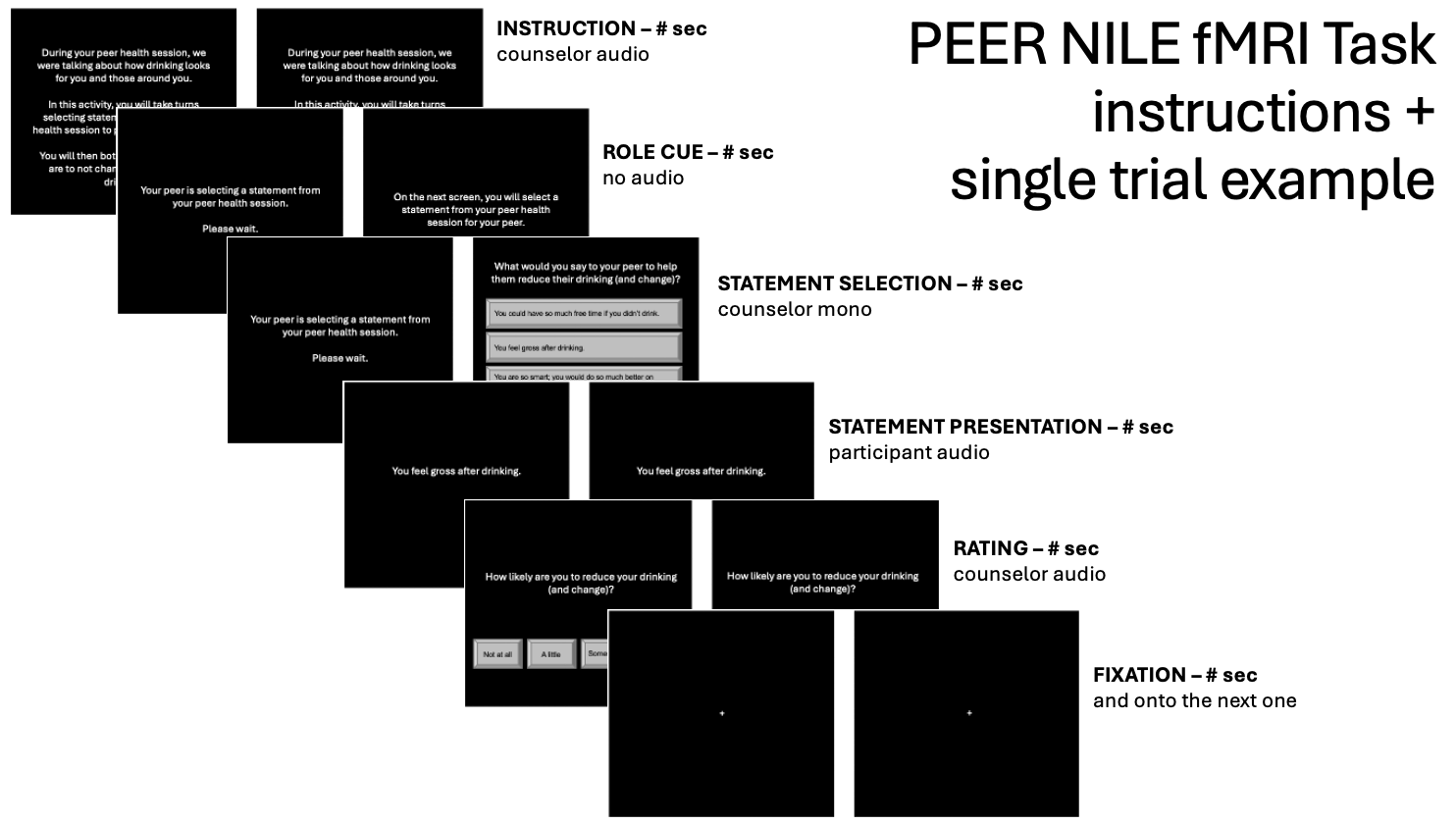


fMRI task (Figure 6). PI Feldstein Ewing and Co-I Filbey have pioneered the examination of within-session factors (client language; therapist language) in the youth brain [2, 3, 59-61]. For this study, we will build directly upon our validated Neural In-Session Language (NILE) Task used in our previous NIAAA-funded translational youth R01 (R01AA023658; project dates 4/17/15- 3/31/22) and published in NeuroImage:Clinical [2]. Our prior study indicated that salient within-session factors during individual-level interventions (there, therapist behaviors) influence the developing brain and that brain response was significantly associated with youth intervention outcomes [2, 3, 59-61]. We will proceed following the same fMRI protocol as that paper. However, different from that study, we will shift the focus from looking at youth response to therapist:youth exchanges within an individual-level intervention (individual MI) and to the current study’s novel context of peer:peer exchanges in a group-level intervention (group MI).

This task is designed to simulate group therapy dynamics by presenting audiovisual statements from dyads recorded during their peer health session. Towards the study aims, fMRI response to peer-generated change talk statements will be measured.

For this task, participating dyads will undergo simultaneous fMRI scans (i.e., hyperscanning) in two adjacent 3T MRI scanners immediately following their virtual peer health session. During the task, each participant will simultaneously be given audiovisual presentations of 4 peer-directed change statements recorded during their peer health session in pseudorandom order. Participants will take turns selecting statements to present to each other.

The task begins with an Instructions screen, with accompanying pre-recorded audio of the peer health session leader, to orient the dyad to the fMRI task. Each trial begins with a Role Cue screen that will designate which participant will make the statement selection, and which will wait while the other makes the selection. This will be followed by a Statement Selection phase where audiovisual stimuli (audio using therapist’s voice and visually presented) will be presented that will ask the designated participant to select a statement from a list of four statements using the button box, while the other participant waits. Next, the participant’s selection will be shown via audiovisual stimuli during the Selection Presentation phase where the selected statement will be delivered visually on the screen in addition to audio recording of the participant (recorded during the health session). Each trial will end with a Rating phase where both participants will be asked to rate how they are to change their drinking behavior via 1 – 4 rating scale (corresponding to a four-button button box) read in the therapist’s voice. There may be a jitter between trials during which a fixation cross will be presented.

Coding fidelity/reliability: PI Feldstein Ewing has been coding client language in MI since 2004 and culling client language for fMRI tasks since 2007. As with PI Feldstein Ewing’s prior NIH-funded studies [2, 3, 59-61], in order to learn how to identify statements for the task, study staff will be required to read the study manual and conduct 5 practice coding sessions by identifying and extracting 4 types of statements in mock interventions conducted by the study team – including in terms of having statements that are sufficiently meaningful (of a sufficient intensity) of the correct valence. This protocol has been highly effective in training study staff to successfully identify and extract statements directly from the recorded MI session and transfer these statements to the fMRI task scan [2, 3, 59-61].

Post-scan check on stimulus delivery: In line with our other studies, following the MRI, U-EA will be asked as part of an exit questionnaire to report the percentage of task statements that they could hear clearly to ensure effective audio delivery and participant attentiveness (cutoff for analysis inclusion = 80%).

**Follow-up Measures**. We will use the same behavioral measurement package and timeline follow back administered at baseline at 3-, 6-, and 12-months to evaluate the nature of U-EAs’ alcohol intervention response. We use a virtual platform for all follow-up assessments [154, 155, 169]. At each follow-up, youth will be emailed unique links to complete validated self-report measures via a secure online data collection system (REDCap) [170], and will complete a brief online or telephone interview with UTD project staff.

Circumstances for withdrawing a subject without their consent

If a subject is unable to keep appointments or follow the researcher’s instructions, including protecting the privacy of the peer health session participant, or is disruptive, uncooperative, threatening or physically violent towards other people during a study visit, their participation will be terminated. If a subject chooses to stop participating for any reason, the youth’s participation will be terminated.

Procedures for withdrawal, including partial withdrawal

If a participant withdraws from the research study, we will continue to retain the data collected up until that point, unless they specifically request removal of their data from the study. In this event, we will destroy all of their data. Subject contact information will be destroyed following the final study participant’s completion of their follow up session; therefore, if a participant requests that their data be removed following the conclusion of the study, we may not be able to complete the request as the data will no longer be identifiable as coming from a particular research subject.

1. Data and Specimens
2. Handling of Data and Specimens

There are two main sources of research data: (1) Participants’ responses for psychosocial, behavioral, and demographic data will be acquired via computerized measures and (2) functional magnetic resonance imaging (fMRI) scans will be taken of each participant while they engage in a behavioral task; both the scans and the response to the behavioral task will serve as measures of brain function and neurocognitive response. The only purpose of the data collected is research. All data after initial entry into the study will be coded based upon subject number, which is not derived from identifying information. The people who will have access to the data include members of the UConn Health/UTD research team. Identifiable data will not be shared with investigators outside of the research team without an executed Data Use Agreement. Participants’ treatment and/or mental health records will not be accessed as part of this study. No invasive procedures are involved in this study.

Consenting materials will be stored electronically on encrypted computers in locked offices, or as paper copies in locked cabinets in locked study offices, depending on how consent forms are completed by the participant. Consent files will be kept separate from participant study materials. UTD, UConn Health, and URI computers are on secure networks, and only lab research staff can access the restricted lab drives. Data collected will be coded with a subject number that does not include any HIPAA identifiers, and only trained UTD research assistants and Co-I Filbey will have access to the password-protected master subject identification sheet linking names with subject numbers. Paper files will be stored in locked cabinets in locked offices, and materials will be labeled only with the assigned study number. Contact information and subject locator forms will be kept separate from subject data, and will be stored as outlined above according to form (paper, etc.). Subject locator forms will be destroyed following the final participant’s completion of the study. Audio recordings will be collected for virtual group MI sessions. Audio recordings will be retained indefinitely. Only IRB-approved research staff will have participant contact and access to identifiable data.

Given that data collection occurs at a subaward site (UTD), the following actions will be taken to secure study data that needs to be accessible to UConn Health and/or URI. First, survey batteries are electronically administered via REDCap, and do not require physical transport; therefore, UConn Health and/or URI study team members can access these data securely through the REDCap portal. Any accompanying paper materials will be in the possession of a UTD study team member at all times, and can be scanned and securely shared electronically if needed. Recordings will be on secure password protected UConn Health, URI, and UTD computers, as group MI sessions are administered virtually by UConn Health or URI study team members connected to a UTD computer, and are directly transferred to the ANCHoR VPN study folder (at UConn Health and URI) and secure UTD lab study folder (at UTD).

Data sharing with approved collaborators (i.e., UTD study team members, or individuals with whom a Data Use Agreement is in place) will take place using an invite-only secure shared folder created by the department IT contact with a built-in expiration (180 days max), or by other university-approved secure sharing processes.

1. Sharing of Results with Subjects

No individual results will be shared. Aggregate results may be disseminated through typical channels (e.g., presentations, manuscripts).

1. Data and Specimen Banking

Study data will be banked for future use, and UConn Health, URI, and UTD IRB oversight will be maintained so long as study data contain HIPAA identifiers. Data released to other investigators outside of the UConn Health, URI, and UTD study teams will be de-identified and labeled only with the subject ID number, unless a Data Use Agreement is obtained. Investigators receiving data will not have access to the key linking subject IDs to participant identities and agree to not attempt to identify any individuals involved in the research study.

1. Data Analysis

Analyses will be conducted to evaluate: (1) changes in brain activation as a result of young people’s own change or sustain language, (2) changes in brain activation as a result of peer-peer dyadic exchanges during group MI, and (3) how brain response relates to youth behavior change. Measures will be derived from the baseline fMRI scan and behavioral data collected at baseline, 3-, 6-, and 12-months.

**MRI preprocessing**. Functional and anatomical MRIs will be processed and analyzed using the Analysis of Functional NeuroImages (AFNI) software package (https://afni.nimh.nih.gov/afni/ version AFNI_22.0.03). The T1-weighted anatomical images will be manually segmented producing a skull-stripped image. A secondary segmentation and warping will then be applied using @SSwarper to the skull-stripped image to refine the segmentation and warp the image to conform to the Montreal Neurological Institute stereotactic space (MNI152 2009). We will generate the functional processing pipeline using AFNI’s afni_proc.py script that discards the first 6 EPI volumes, implements slice timing and field map distortion corrections, registers fMRI volumes to the minimum outlier, aligns and warps volumes to the template space provided by the anatomical transformation, spatially smooths using a Gaussian filter (5 mm full-width-at-half-maximum), and mean scales voxels time series to 100. Frame-to-frame displacement will be calculated for each volume.

**First-level univariate models**. For first-level univariate models, model fitting will be computed with the 3dDeconvolve and SPMG2 basis functions, as well as 3dREMLfit estimation of auto-correlation. Stimulus timing files from the fMRI task will be entered into models as well as nuisance regressors estimating frame-to-frame translational and rotational motion and signal intensity outliers.

**Regions-of-Interest (ROIs)**. ROIs will be defined using the probabilistic Harvard-Oxford cortical and subcortical structural atlases (distributed with FSL; https://fsl.fmrib.ox.ac.uk/fsl/fslwiki/Atlases). ROIs will be constructed as 5mm radius spheres.

**Behavioral analyses**. We will leverage the dynamic nature of the PEER hyperscanning task to examine neural responses in the U-EAs social cognition network (medial prefrontal cortex [mPFC], superior temporal sulcus [STS], temporoparietal junction [TPJ]) as participants engage with a novel peer from their group MI session. We will examine the degree to which synchrony of BOLD response that occurs while hearing and seeing prosocial peer-directed health promotive language (peer-directed change talk) directly generated during and extracted from the group MI session is associated with youth behavior change (past month drinking days) at 12 months post-intervention. It is expected that the BOLD synchrony during peer-directed change language will be associated with behavior change at 12 months. Primary outcomes include behavioral measures (TLFB) to assess past month drinking days at 12 months post-intervention. Secondary outcomes are the same behavioral measures collected at 3 and 6 months. A linear mixed‑effects model will be used to examine how fMRI‑based hyperscanning metrics—specifically, synchrony of BOLD responses within the social cognition network—predict changes in behavior over time (129, 130). In this model, BOLD synchrony will be included as a time‑invariant predictor, measurement occasion (3, 6, and 12 months) as a categorical time‑varying predictor, and behavioral outcomes as repeated measures, allowing simultaneous evaluation of the relationship between the BOLD synchrony during the intervention and subsequent behavioral changes at three time points.

Inter-brain synchrony will be quantified to capture moment-to-moment coordinated neural responses between each dyad during the hyperscanning fMRI task. Mean BOLD time series will be obtained for each ROI and temporally aligned across participants. Inter-brain synchrony will be quantified using Pearson correlation coefficients (r) between the BOLD time series of dyad members within ROIs encompassing the U-EA social cognition network (mPFC, STS, TPJ). To capture moment-to-moment neural alignment, correlations will be computed using a sliding-window approach, with window size and step size selected to balance temporal resolution and signal stability. Correlation values will be Fisher Z-transformed prior to group-level analyses to ensure normality.

These synchrony metrics (i.e., ROI z-scores) will be used to test our hypothesis that greater neural alignment in the social cognition network during prosocial peer-directed change language will be associated with greater behavior change. Permutation testing and multiple comparison correction will be applied when assessing statistical significance.

*Missing data*

Linear mixed‑effects models using restricted maximum likelihood estimation (REML) will handle missing behavioral data over time, under the realistic assumption that data are missing at random—that is, missingness at a given time point depends on observed covariates and on outcomes measured at earlier time points (131).

1. Privacy, Confidentiality and Data Security

Prior to participation, participants are informed of all study procedures by UTD staff members, and must proceed through an informed consent process stating that they understand and agree to the procedures. This helps ensure that those who enroll are comfortable with the study procedures and the research topics of interest (i.e., health promotion and related health risk behaviors).

Consenting materials will be stored electronically on encrypted computers in locked offices, or as paper copies in locked cabinets in locked UTD study offices, depending on how consent forms are completed. Consent files will be kept separate from participant study materials. UConn Health and UTD computers are on secure networks, and only research staff can access the restricted lab drives. Data collected will be coded with a subject number that does not include any HIPAA identifiers, and only trained UTD research assistants and Co-I Filbey will have access to the password-protected master subject identification sheet linking names with subject numbers. Paper files will be stored in locked cabinets in locked offices, and materials will be labeled only with the assigned study number. Contact information and subject locator forms will be kept separate from subject data, and will be stored as outlined above according to form (paper, etc.).

All study staff will complete the required institution-specific training modules for clinical trials research with human subjects, and be trained on lab and study-specific protocols to protect participant confidentiality.

Given that data collection occurs at a subaward site (UTD), the following actions will be taken to secure study data that needs to be accessible to UConn Health. First, survey batteries are electronically administered via REDCap, and do not require physical transport; therefore, UConn Health and URI study team members can access these data securely through the REDCap portal. Any accompanying paper materials will be in the possession of a UTD study team member at all times, and can be scanned and securely shared electronically if needed. Recordings will be on secure password protected UConn Health, URI, and UTD computers, as group MI sessions are administered virtually by a UConn Health and/or URI study team member connected to a UTD computer, and are directly transferred to the ANCHoR VPN study folder (at UConn Health and URI) and secure UTD lab study folder (at UTD).

Data sharing with approved collaborators (i.e., UTD study team members, or individuals with whom a Data Use Agreement is in place) will take place using an invite-only secure shared folder created by the department IT contact with a built-in expiration (180 days max), or by other university-approved secure sharing processes.

1. Risks and Benefits
2. Risks to Subjects

There are five potential risks associated with participation and every effort will be made to reduce potential discomfort. **First**, there is the potential discomfort involved in answering questions about sensitive topics, including alcohol use behavior. **Second**, there is the risk of breach of confidentiality. **Third**, while the fMRI procedures involve no pain or invasive techniques, individuals occasionally find the partially enclosed space and/or the noise of the scanner uncomfortable. Adverse events associated with fMRI can involve the action of the magnetic field on metallic objects. For example, metallic objects on or inside the body of participants or foreign metallic objects that are brought into the room with the magnet may injure participants. **Fourth**, standard risks for youth health risk behavior research often include discussions regarding illegal activities (e.g., discussions about drinking, as alcohol use under the age of 21 is illegal). **Fifth**, another standard risk includes confidentiality concerns during group intervention sessions. However, in line with the PI’s prior clinical work and clinical research with group interventions, this risk will be minimized through active established efforts to protect the confidentiality of all youth during group intervention sessions. Specifically, dyads will be rescheduled if they are inadvertently assigned to group MI with a known individual.

As has been done in the PI’s prior research, these risks will be minimized through active efforts detailed here. **First**, prior to participation, participants are informed of all procedures, and they must proceed through an informed consent process and provide consent stating that they understand and agree to the procedures. Therefore, it is likely that any individual who would be made uncomfortable by answering questions about alcohol use behavior would decline to participate. **Second**, to ensure youth’s confidentiality, all data will be identified with a numeric code and stored separate from consent forms and confidential locator forms. In addition, UE-As’ data will be retained by the PI and lab manager, and maintained separately. The confidential locator forms will be destroyed upon participants’ completion of the final follow-up. **Third**, our staff and UTD magnetic resonance (MR) technicians are highly experienced and have received specific training to prevent accidents in the fMRI scanner. Both the trained study staff and the MR technician provide a rigorous screening to ensure that participants do not have any metal on or inside their bodies prior to entering into the scanner. This screening includes a thorough metal screening form covering possible procedures, implants, and exposures that may impact the safety and data quality of the MRI experience. In addition, access to the magnet is limited to prevent others from bringing in metallic objects. We also screen for claustrophobia and provide individuals with ear protection to reduce the amount of noise they experience. It is still possible that individuals may feel anxious in the scanner, but they are in constant communication with the experimenters (i.e., through headphones and microphone) and can stop the experiment (i.e., using an emergency bulb) and be removed at any time if they are feeling too anxious to continue. If the researcher and/or the MRI Technologist observes something in any scan that might be an indication of an abnormality, the procedures in the Center for BrainHealth Incidental Findings Checklist will be followed. The technologist will forward the scan(s) to a consulting radiologist for evaluation. The radiologist will provide a report to the Imaging Center staff, which will then be forwarded to the PI. The PI of each study has primary responsibility to review and follow the radiologist’s guidance. If the radiologist indicates that the finding is potentially significant, the PI shall report this adverse event to the IRB and notify the participant of the radiologist’s guidance. No diagnosis should be provided as part of the participant notice. For participants who suspect they may be pregnant at the time of scanning, we have pregnancy tests available to verify status, and will not proceed with scanning pregnant individuals. **Fourth**, we have not encountered any reticence by the youth to disclose their substance use and related risk behavior. Completion of the assessment measures has minimal risk and youths are notified that they may skip questions that are distressing for them to answer without it negatively impacting their compensation or status within the study. **Fifth**, our team is highly experienced at working with youth in clinical work and clinical research with group interventions, including who are engaging in illegal behaviors, such as substance use. We will continue to take exhaustive steps to protect all youth participants’ privacy in this study.

1. Potential Benefits to Subjects

This study is expected to add critical data that will add to the knowledge base to improve alcohol use treatment for U-EA engaged in hazardous alcohol use. Given the minimal risk to participants and the greater possibility of long-term benefit to the participant and the greater knowledge base, the risk/benefit ratio seems reasonable. As a part of this study, all participants will have the opportunity to examine their own alcohol use behavior in the context of completing measurement instruments, have the opportunity to work with a trained therapist, and receive a behavioral alcohol treatment, which may help facilitate their efforts to reduce their hazardous drinking. The costs associated with participating have been minimized via the consent procedures, procedures for maintaining confidentiality, and safeguards during the fMRI component. The minimal costs associated with participation in this research are reasonable in relation to the anticipated benefits to the participants themselves. In terms of benefits to others, evaluating neural and behavioral impact of group MI on U-EA hazardous drinking and proposed neurodevelopmental mechanisms has a great potential to benefit others.
